# The burden of disease in patients eligible for mentalization-based treatment (MBT): quality of life and costs

**DOI:** 10.1186/s12955-016-0538-z

**Published:** 2016-10-12

**Authors:** Elisabeth M. P. Laurenssen, Hester V. Eeren, Martijn J. Kikkert, Jaap Peen, Dieuwertje Westra, Jack J. M. Dekker, Jan J. V. Busschbach

**Affiliations:** 1 Viersprong Institute for Studies on Personality Disorders (VISPD), Halsteren, The Netherlands; 2 Arkin, Amsterdam, The Netherlands; 3 De Viersprong, Amsterdam, The Netherlands; 4 Department of Clinical Psychology, VU University Amsterdam, Amsterdam, The Netherlands; 5 Department of Psychiatry, Section Medical Psychology and Psychotherapy, Erasmus MC, Rotterdam, The Netherlands

**Keywords:** Quality of life, Costs, Borderline, Mentalization-based treatment

## Abstract

**Background:**

Mentalization-Based Treatment (MBT) is a promising, though expensive treatment for severely ill patients with Borderline Personality Disorder (BPD). A high burden of disease in terms of quality of life (QoL) and life years lost can be a reason to prioritize mental health interventions, and specifically for BPD patients. Moreover, when the societal costs of the illness are high, spending resources on high treatment costs would be more easily legitimized. Therefore, the purpose of this study was to calculate the burden of disease of BPD patients eligible for MBT.

**Methods:**

The 403 patients included in this study were recruited from two mental health care institutes in the Netherlands. All patients were eligible for MBT. Burden of disease consisted of QoL, measured with the EuroQol EQ-5D-3L, and costs, calculated using the Trimbos and Institute for Medical Technology Assessment Questionnaire for Costs Associated with Psychiatric Illness.

**Results:**

The mean QoL index score was .48. The mean total costs in the year prior to treatment were €16,879 per patient, of which 21 % consisted of productivity costs.

**Conclusions:**

The burden of disease in BPD patients eligible for MBT is high, which makes it more likely that society is willing to invest in treatment for these patients. However, this finding should not be interpreted as a license to unlimitedly use resources to reimburse treatment for severe BPD patients, as these findings do not provide any information on the effectiveness of MBT or other available treatment programs for BPD. The effectiveness of available treatments should be evident by studies on the effectiveness of the treatment itself and by comparing the effectiveness of these treatments to treatment as usual and to other treatment options for BPD patients.

**Trial registration:**

The data on this paper came from two trials: NTR2175 and NTR2292.

## Background

Borderline personality disorder (BPD) is one of the most prevalent mental disorders in psychiatric populations [1, 2]. Mentalization-based treatment (MBT) is often the treatment of choice for patients with a severe BPD, as MBT claims to be able to treat BPD patients situated at the more severe end of the continuum of severity of pathology [3]. MBT aims to enhance patients’ mentalizing capacity, particularly in high arousal contexts. Mentalizing refers to “the mental process by which an individual implicitly and explicitly interprets the actions of himself and others as meaningful on the basis of intentional mental states such as personal desires, needs, feelings, beliefs, and reasons” [4]. In MBT, impairments in mentalizing are believed to be a core feature of patients with BPD, and are related to problems with affect regulation and attentional control. Hence, improving this capacity is thought to be associated with a decreased need to rely on maladaptive coping strategies to deal with feelings of inner emptiness, impulsivity and conflicts in interpersonal relationships. As a consequence, this decreases symptoms and enhances interpersonal functioning, which is a treatment goal of MBT [4].

Besides being a promising treatment for severely disordered BPD patients, MBT is also considered an expensive treatment, as the treatment intensity is high and MBT is given by only highly specialized therapists [4]. Given the high costs of treatment, it is warranted to find evidence to prioritize such an intervention for severe BPD patients. A high burden of disease in terms of quality of life (QoL) and life years lost can be a reason to prioritize health interventions aimed at improving the health status of these patients [5]. The willingness to pay for such treatment programs tends to be higher than for patients having less severe health conditions [5]. This relation between burden of disease and willingness to pay has also been imbedded in economic theory, usually under the name ‘equity’ or ‘solidarity’ [6] and is tested in panels of policy makers and lay people [7–9]. It explains why we value a small health gain in severely ill patients higher than the same health gain in patients with a relatively low burden of disease. For instance, in the United Kingdom, the willingness to pay ‘thresholds’ for treatments are higher for patients in the last phase of their life compared to patients who still have a substantial life expectancy [7]. New standards on cost-effectiveness in the Netherlands also recognize different thresholds which are related to the burden of disease: the higher the burden of disease, the higher the willingness to pay thresholds for treatments [10], while for example in Belgium, the out of pocket co-payment of patients is lower when the burden of disease is higher (http://www.bcfi.be/nl/start). In addition, people not only tend to put a high value on interventions for severe health conditions [5], and are more willing to allocate resources to treat those patients, they also accept a higher cost-effectiveness ratio when patients suffer from a high burden in terms of QoL and costs [5]. Moreover, when the costs of disease for society are high, it is more likely that an effective treatment will accomplish cost savings for society. Thus, for a severely ill patient group, it is important to estimate their burden of disease, as this burden of disease is a criterion on which to prioritize health interventions for these patients. The burden of disease is often presented as QoL and societal costs. For BPD patients in general, the QoL and societal costs are estimated in some earlier studies, on which we will elaborate first.

Several studies found that patients with BPD suffer from a low QoL, as measured with the generic EQ-5D [11], where the QoL index score as measured with the EQ-5D is expressed as a single index score, where a score of 1 represents the value of perfect health, and a score of 0 represents the value of death. In a Dutch study the QoL index score was calculated in a sample of 1708 patients with personality disorders (PDs) and it was estimated to be .52 in BPD patients, representing a severe burden of disease [12]. Van Asselt and colleagues [13] performed a randomized controlled trial (RCT) in BPD patients comparing schema-focused therapy (SFT) and transference-focused psychotherapy (TFP). They found baseline QoL index scores of .49 for the SFT group and .46 for the TFP group. McMain and colleagues [14] calculated the baseline QoL index score of 180 BPD patients referred to either dialectical behavior therapy (DBT) or to general psychiatric management to be .57 and .55, respectively. And though the sample was small, Bales and colleagues [3] found a QoL index score of .49 in patients allocated to MBT. Moreover, the QoL in patients with BPD is comparable to that of patients with severe physical conditions, such as stroke and Parkinson’s disease (QoL index score of .49 and .44, respectively) [15, 16] and it is lower than a severe mental disorder such as major depressive disorder (QoL index score of .58) [17]. In comparison to the mean QoL index score of the general population in Western societies, which ranges between .83 and .87 [18–20], the QoL of BPD patients is low.

In addition to a low QoL, BPD also causes a high economic burden on society. Van Asselt and colleagues [21] found the total costs per BPD patient to be €16,852 in the year prior to treatment. Soeteman and colleagues [22] assessed the economic burden of PDs in 1740 patients with personality pathology. They found the total cost of patients with a PD in the year prior to treatment to be estimated at €11,126 per patient. BPD was uniquely associated with an increased mean total costs. Though not taking into account the indirect costs due to productivity losses, such as absence from work or reduced efficiency at work, Bateman and Fonagy also gave an indication of the economic burden of patients allocated to MBT [23]. They estimated the health care utilization costs for BPD patients receiving psychoanalytically oriented partial hospitalization in the 6 months before randomization to be $2379 (€2141; adjusted for inflation and purchasing power parities [24]).

Thus, published evidence presents a low QoL in BPD patients in general, and high societal costs. However, to our knowledge, the QoL in BPD patients eligible for MBT was only estimated in a small sample by Bales [3] and the costs in BPD patients eligible for MBT was only estimated by Bateman [23]. More research is clearly needed, particularly as patients with BPD referred to MBT tend to be patients situated at the more severe end of the continuum [3]. The aim of the present study was therefore to fill this gap of knowledge for these severe BPD patients. Because of the severity of problems of these patients, we expected these BPD patients to have lower QoL and higher costs than other (B)PD patients. In this study we estimate the burden of disease of BPD patients eligible for MBT in term of QoL and societal costs, by combining baseline data of two RCTs in the Netherlands.

## Methods

Data for this study were collected from two RCTs in the Netherlands. In the first RCT, called MBT-TAU, Day Hospital MBT (MBT-DH) was compared to a specialist Treatment As Usual (S-TAU) and was performed at two mental health care centres in Amsterdam: Arkin and De Viersprong [25]. The second RCT, called MBT-DOS, compared MBT-DH with MBT Intensive Outpatient treatment (MBT-IOP) and was executed in Amsterdam (Arkin and De Viersprong), Bergen op Zoom (De Viersprong) and Groningen (Lentis) [26]. All treatment centres were specialized in treating BPD patients. The protocols of both trials were described in detail elsewhere [25, 26].

### Patients

In total, 403 patients were included between March 2009 and July 2014. Patients were included in these studies when they had a BPD diagnosis as measured by the Structured Clinical Interview for DSM-IV Axis II Personality Disorders (SCID-II) [27]. In the MBT-TAU study, patients should also have a total score on the Borderline Personality Disorder Severity Index (BPDSI) [28] of at least 20, reflecting severe BPD. Exclusion criteria were: (a) the presence of an Axis-I disorder (as determined with the Structured Clinical Interview for DSM-IV Axis I Personality Disorders (SCID-I) [29] that required specialist treatment, (b) a diagnosis of autism spectrum disorders or organic brain disorder that interferes significantly with the ability to mentalize; (c) IQ below 80 as measured by the Wechsler Adult Intelligent Scale–III [30], (d) inadequate mastery of the Dutch language, and (e) a diagnosis of antisocial personality disorder in combination with a history of severe physical violence [25, 26].

### Procedure

The SCID-I and SCID-II interviews were administered as part of the formal admission procedure (De Viersprong) or to assess whether patients were eligible for the trial (Arkin and Lentis). If patients fulfilled the inclusion criteria for one of the trials, patients signed informed consent and were asked to complete a baseline assessment, including the EQ-5D-3L and TiC-P measurements. In the MBT-DOS trial, patients completed the baseline assessment before randomization. In the MBT-TAU study, patients completed the baseline assessments partly before and partly after randomization.

### Measures

#### Axis I and Axis II disorders

Axis I disorders were diagnosed by using the Structured Clinical Interview for DSM-IV Axis I disorders (SCID-I) [29, 31]. The Structured Clinical Interview for DSM-IV Axis II Personality disorders (SCID-II) [27, 32] was used for diagnosing Axis II personality disorders. Criteria were scored if they were pathologic, pervasive, persistent and if they were present for at least 5 years, consistent with the DSM-IV-TR general diagnostic criteria for the presence of a personality disorder [33].

### Symptoms

The Dutch version of the Brief Symptom Inventory (BSI) [34, 35] was used to assess general psychopathological symptoms. The BSI is the short version of the Symptom Checklist-90. It includes 53 items covering nine symptom dimensions (somatization, obsession-compulsion, interpersonal sensitivity, depression, anxiety, hostility, phobic anxiety, paranoid ideation and psychoticism) and yields three global indices of distress: Positive Symptom Distress Index, Positive Symptom Total, and Global Severity Index (GSI). Possible GSI scores range from 0 to 4, with higher scores indicating a higher level of psychological and emotional distress. Respondents have to rate each item (e.g. “your feelings are easily hurt”) on a five-point scale ranging from 0 (not at all) to 4 (extremely), representing the intensity of distress relating to each item during the past 7 days. The reliability of the Dutch version of the BSI is good (Cronbach’s α ranging from .71 to .88, test-retest reliability ranging from *r* = .71 to .89). These values are comparable to the original BSI version of Derogatis [34].

### Quality of life

QoL was measured using the EuroQol EQ-5D-3L [36]. This self-report questionnaire provides a simple method to measure health problems in 5 dimensions: mobility, self-care, usual activities, pain/discomfort and anxiety/depression. Each dimension is divided into three response-levels: no problem, moderate problems, and extreme problems. Combining these scores gives 243 possible health states, where each of these is weighted to obtain a single index score between -.33 (worst imaginable health state) and 1 (best imaginable health state) [37]. A score of 1 represents the value of perfect health, whereas a score of 0 represents the value of death. Sometimes patients experience extreme problems on all five dimensions, which can turn their QoL index score below zero. However, normally this state is temporary [38]. To calculate the mean QoL index score, we used norm scores of a Dutch validation study [37]. The QoL index score is thus estimated based on the preferences of the Dutch general public and represents a value the public would assign to the measured health state [39]. The reliability of the EQ-5D-3L has been investigated and found to be acceptable [40] and it has shown to be sensitive to change in patients with personality disorders [22, 41].

### Costs

The direct costs (e.g. increased health care utilization) and indirect costs (e.g. productivity losses due to disability and to higher absence from work) in the year prior to treatment were assessed using the Trimbos and Institute for Medical Technology Assessment Questionnaire for Costs Associated with Psychiatric Illness (TiC-P) [42, 43]. The first part of the TiC-P consists of questions on (1) the number of visits to, e.g. a general practitioner, psychiatrist (outside the treatment offered in the trials), medical specialist, physiotherapist or alternative health practitioner; (2) the day care/hospital lengths of stay (outside treatment offered in the trials); and (3) the use of medication in the 4 weeks prior to the administration of the questionnaire in MBT-DOS and 6 months in MBT-TAU [42]. These care and cure volumes were multiplied with unit prices of the corresponding health care services according to the Dutch manual for costing studies in health care [44]. The unit prices for 2010 will be adjusted to 2014 prices using the Consumer Price Index [45]. Both recall times (4 weeks in MBT-DOS and 6 months in MBT-TAU) were standardized to 1 year.

The second part of the TiC-P asks the patient to report absence from work, reduced efficiency at work and difficulties with nonpaying jobs. The days of short-term absence from work and actual hours missed at work due to health-related problems were multiplied with the average productivity costs per employee per hour, taking into account the number of days and hours of paid employment of the patient per week. Because the recall period of this part of the TiC-P was 2 weeks, we multiplied the costs by 26 to estimate the annual costs. To value long-term absence from work, we applied the friction cost method [22, 46, 47]. This method takes the employer's perspective, and counts as lost only those hours not worked until another employee takes over the patient's work. This period, the so-called friction period, is estimated to be 116 days. Hence, the maximum indirect costs were limited to productivity losses during 116 days. The friction cost method is a more conservative estimate than the so-called “human capital method”, which relates productivity costs one-to-one to the labour costs of the patient. The choice between friction costs and human capital is still a subject of debate among economists [22, 46, 47]. Furthermore, difficulties with non-paying jobs were estimated as hours a patient was supported in for example housekeeping activities, and was multiplied with the corresponding unit prices [42, 43].

### Burden of disease

We estimated the burden of our patient population in terms of their QoL and direct and indirect costs, which represent the societal costs of these patients. In addition, we described their socio-demographic and diagnostic data and GSI scores. We reported the mean and standard deviation of the QoL index score, the direct and indirect costs, and the GSI score.

## Results

### Participants

Table 1 presents the social demographic and diagnostic data at baseline. As can be expected, the majority of the sample was female (82 %). The majority (81 %) of the patients had at least one co-occurring Axis-I disorder and 41 % had at least one co-occurring Axis-II disorder. The mean GSI score at baseline was 1.92 (SD = .79); representing a high level of psychological and emotional distress.

**Table 1:** Sociodemographic and diagnostic data

	Number	Percent	Valid N	Missing
Gender (Male)	71	17.6	403	0
Self-harm in the past 6 months	150	45.2	332	71
Suicide attempt in the past 6 months	125	37.3	335	68
*Co-morbid Axis I disorders*				
Anxiety disorder	184	46.6	395	8
Mood disorder	229	57.8	396	7
Pychotic disorder	16	4.0	396	7
Eating disorder	90	22.7	396	7
At least 1 co-morbid Axis I disorder	320	80.8	396	7
*Co-morbid Axis II disorders*				
Avoidant PD	62	15.4	403	0
Obsessive compulsive PD	24	6.0	403	0
Histroic PD	2	.5	403	0
Narcissictic PD	10	2.5	403	0
At least 1 co-morbid Axis II disorder	164	40.7	403	0
	Mean	SD	Valid N	Missing
Age	31.37	9.47	344	59
QoL index score	.48	.29	316	87
GSI score	1.92	.79	335	68

*QoL index score* Quality of Life index score, *GSI* Global Severity Index

### Burden of disease: quality of life and costs

The mean QoL index score in our patient population was .48 (SD = .29), representing a severe burden of disease (Table 1). Table 2 describes the direct and indirect costs in the year prior to treatment. The mean total direct costs per patient were €13,405 per year. Those costs were mainly composed of costs due to inpatient health care (39.1 %), outpatient mental health care (18.6 %) and day hospital care (14 %). The total mean indirect costs per patient were €3474 per year. Here, absence from work and reduced efficiency at work were estimated for the 90 patients (28.1 % of 320 valid N; 83 missing values) that had a paying job. These patients reported the total hours lost to be 101.34 (Table 2). The mean total costs of BPD patients were €16,879 per year.

**Table 2:** Mean costs per year of patients with BPD

Type of service	Mean quantity	Mean cost (2014 prices), €	SD cost (2014 prices), €	Percentage of total direct medical costs	Subjects using the service, N	Valid N	Missings	Subjects using the service, (% of valid N)
*Direct medical costs per year*								
General practitioner	10.16	312.70	636.93	2.3	141	322	81	43.8
Company doctor	2.07	63.56	163.38	.5	56	308	95	18.2
Physiotherapist	3.54	140.26	644.45	1.0	30	317	86	9.5
Alternative health practitioner	2.15	116.45	772.13	.9	21	320	83	6.6
Domestic help	44.34	704.33	3235.95	5.3	47	339	64	13.9
Self help group	4.7	271.76	1732.54	2.0	19	315	88	6.0
Social worker	3.55	253.46	960.62	1.9	47	315	88	14.9
Substance abuse outpatient care	1.29	39.53	286.92	.3	18	319	84	5.6
Outpatient mental health care	13.24	2488.36	5589.44	18.6	113	288	115	39.2
Psychiatric practice	9.8	947.85	2175.99	7.1	95	296	107	32.1
Outpatient clinic	2.84	359.08	1453.43	2.7	36	310	93	11.6
Day hospital care	10.39	1880.12	6779.94	14.0	44	314	89	14.0
Inpatient health care	11.4	5239.60	27586.25	39.1	28	314	89	8.9
Medical specialist	1.94	153.48	510.58	1.1	41	313	90	13.1
Medication	NA	434.48	634.35	3.2	82	109	294	75.2
*Total*		*13405.00*						
*Indirect costs per year*								
Absence from work^a^	84.14	2502.54	10005.20	72.0	41	317	86	12.9
Reduced efficiency at work^a^	17.3	467.24	2951.80	13.4	16	283	120	5.7
Difficulties with nonpaying jobs	36.73	504.63	1466.64	14.5	44	241	162	18.3
*Total*		*3474.41*						

*NA* Not Applicable

^a^Estimated for 90 patients that had a paying job (320 valid N; 83 missing values)

## Discussion

BPD patients eligible for MBT reported a severe QoL and were accountable for high costs on society. The main cost drivers were inpatient mental health care, outpatient mental health care and absence from work.

The QoL in our sample seems lower or at least comparable to patients with severe physical and mental illnesses, for example diabetes (QoL index score of .62) [15], asthma (QoL index score of .67) [15], ischaemic heart disease (QoL index score of .55) [15], chronic pain with neuropathic characteristics (QoL index score of .47) [48], Parkinson’s disease (QoL index score of .44) [16] and major depressive disorder (QoL index score of .58) [17]. The mean QoL index score in our sample is also comparable to the QoL index score in BPD patients estimated in other studies, with QoL index score ranging from .46 to .57 [3, 12–14]. This result is in contrast to our expectation that BPD patients eligible for MBT patients would have lower QoL than other (B)PD patients.

The economic burden of BPD patients eligible for MBT seems considerably higher than the burden of patients seeking mental health treatment for other mental and physical disorders, such as anxiety disorders, mood disorders and Parkinson’s disease, which cost €1077, €3406 and €11,153 per patient per year respectively [49]. The economic burden in our trial is comparable to the costs of psychotic disorders: €18,796 [49] and to two other studies on costs of PDs and BPD: €11,126 per patient in the study by Soeteman [22] and €16,205 in patients with BPD per year in the study by Van Asselt and colleagues [21]. The costs that Bateman and Fonagy calculated in the 6 months before randomization were $2379; when adjusted for inflation and purchasing power parities, costs per person per year were approximately €2141 [24]. These costs are substantially lower than the total costs in our trial, which is probably due to the exclusion of indirect costs in their analyses [23]. Wagner and colleagues [50] calculated that in the year prior to dialectical behavior therapy, BPD patients had a total mean annual societal cost of €28,026 per patients. This is more than €10,000 higher than the societal costs we found in our study (€16,879). This difference could be explained by using a different method to estimate productivity losses. Wagner and colleagues used the Human Capital Approach (HCA) [51], whereas we used the Friction Cost Method [52]. Using the HCA, it is assumed that productivity losses last until the age of retirement or until the time the person has found an equivalent job. The friction cost method estimates productivity losses in a more conservative manner by using the time needed to replace a worker. This method is believed to result in actual productivity costs, whereas HCA is criticized for calculating potential rather than actual productivity costs, leading to unrealistically high estimates of productivity costs [22, 53]. The finding that the costs in our patient population was comparable to other BPD patients and other PD populations was not in line with our expectations; we expected severe BPD patients eligible for MBT to report higher societal costs than other patient populations.

### Strengths and limitations

One of the strengths of this study is the use of a generic QoL measure, which made it possible to compare the QoL index score of the BPD patients in our study to that of patients with other mental and physical illnesses. Although it is suggested that the EQ-5D-3 L may not be sensitive enough to reflect the impact of severe mental disorders, such as chronic schizophrenia and PDs [54], the current study showed that these concerns are not justified for severe BPD patients in our study population. We found a severely disturbed QoL in these patients, which indicated that an important part of the problems in this particular patient group was well captured with the EQ-5D-3 L. In addition, other studies on QoL in BPD patients found similar QoL index scores, indicating the robustness of the present findings [12, 55]. A second strength was the use of an extensive cost questionnaire, by which we could calculate both direct and indirect costs. Bateman and Fonagy only estimated direct costs, and did not take into account the indirect costs due to productivity losses [23]. The current study now added those costs to the total costs of patients eligible for MBT, and made costs comparable to other studies on the economic burden of PD in general and BPD specifically.

We only included BPD patients eligible for MBT, which may limit the generalizability of our study results to the general group of BPD patients. However, our results showed that societal costs and QoL were comparable to other studies on QoL and costs in BPD patients. A second limitation is the considerable number of missing data, especially in the cost data, as can be seen in Table 2. We mostly missed data from the RCT that aimed to compare MBT-DH with S-TAU. This could be explained by the procedure of measurements. Only the SCID-II and the BPDSI were calculated for all patients before randomization as part as the admission procedure for this study. The other baseline measures were administered after randomization, including the EQ-5D-3L, TiC-P and BSI. As a lot of the S-TAU patients were unhappy with their treatment allocation, many of these patients refused to complete the test battery after randomization. Though other studies on costs in BPD patients do not mention missing data [21–23], we know out of personal communication that the study by Soeteman also had a considerable number of missing data. Despite the missing data in our trial, the QoL and EQ-5D score were still within the same range as these earlier studies. Missing data influences, however, the costs estimates as presented in Table 2. On the one hand, the patients whose costs were unknown could have had a high amount of health- related costs, such that the overall estimated costs represent an underestimation. On the other hand, it is possible that these patients had no costs at all. In that case, the estimated costs in this study could be overestimated, because we only represented the costs of those patients that actually filled in the cost questionnaire. A third limitation relates to the measurement of costs by self-report. The costs were not based on more objective sources such as data from health insurance companies, which may have led to an underestimation of costs. However, self-report was commonly used to calculate costs on BPD patients in earlier studies, and to our knowledge, there are no studies using data from health insurance companies yet. In addition, data from health insurance companies is limited to costs covered by the health insurance and has no information on, for example, out of pocket costs. A fifth limitation is that we calculated the costs prior to start of treatment. These costs may be very high, because patients had severe problems, for which they eventually received treatment. On the other hand, it is also possible that costs prior to treatment are lower than usual, because patients know they will receive treatment and therefore don’t make use of other resources.

### Practical implications

Our study showed a high burden of disease in BPD patients eligible for MBT. This high burden can help prioritizing health interventions for severely ill BPD patients in general, and MBT specifically, as people tend to be willing to pay more to treat patients with a higher burden of disease. Given the low QoL and high societal burden we found in this study, an expensive treatment for severely ill BPD patients, such as MBT, can more easily be legitimized. However, as the burden of disease in severe BPD patients is comparable to the burden of other BPD patients and patients with other PDs, prioritization of resources for severe BPD patients based on (cost)-effectiveness research becomes even more important. Though an expensive treatment is more easily legitimized, our results provide no license to unlimitedly use resources to reimburse MBT or other highly specialized treatments for severe BPD patients, as these findings do not provide any information on the (cost-)effectiveness of available treatment programs. The effectiveness of treatment programs should be evident, not only by studies on the effectiveness of the treatment itself, but also by comparing the effectiveness of MBT to TAU and to other highly specialized treatments for BPD patients. Although there is some promising evidence supporting the effectiveness of MBT-DH and MBT-IOP [3, 56–58], given the small number of studies, more research is urgently needed, particularly in light of the limitations of existing trials. These include possible researcher allegiance, questions about generalizability and the lack of a credible TAU. Moreover, more research into the effectiveness of other specialized treatments for BPD, such as SFT and DBT, is also warranted. Various studies show that the effectiveness of these highly specialized treatments seems comparable to well-specified treatments for BPD that are delivered in a consistent, coherent and continuous way [59–61]. As a result, in practice, integrative treatments are already being developed [62], combining the best of specialist techniques and common factors. Yet, research on the effectiveness of these integrated treatments is needed.

Furthermore, when for example MBT turns out to be more effective, but also more costly than the intervention to which it would be compared, a cost-effectiveness analysis of MBT is needed to estimate the cost-effectiveness ratio of MBT compared to alternative treatment options. Currently, we are comparing day hospital MBT to specialist TAU [25] on its effectiveness and costs. At the same time, two intensities of MBT, day-hospital MBT [56] and intensive outpatient MBT [57], are compared on effectiveness and costs [26]. Cost-effectiveness analyses can support the reimbursement decision in which treatment should be paid for. However, a cost-effectiveness analysis is not the only tool to decide on this. The burden of disease is also an important criteria to decide which treatment should be paid for, and can yield a strong argument in favor of reimbursing treatments for severely ill BPD patients.

## Conclusions

Given the low QoL and high societal burden we found in this study, it can be more easily legitimized to treat severely ill BPD patients with an expensive treatment such as MBT. However, more research is needed on the (cost)-effectiveness of specialist treatments for BPD such as MBT versus TAU, integrated treatments and other specialist treatments for BPD, as the findings from this study do not provide any information on the effectiveness of available treatment programs.

## Abbreviations

BPD: Borderline personality disorder
BPDSI: Borderline personality disorder severity index
BSI: Brief symptom inventory
DBT: Dialectical behavior therapy
GSI: Global severity index
HCA: Human capital approach
MBT: Mentalization-based treatment
MBT-DH: Day hospital mentalization-based treatment
MBT-IOP: Intensive outpatient mentalization-based treatment
PD: Personality disorder
QoL: Quality of life
RCT: Randomized controlled trial
SCID-I: Structured Clinical Interview for DSM-IV axis I Personality Disorders
SCID-II: Structured Clinical Interview for DSM-IV Axis II Personality Disorders
SFT: Schema-focused therapy
S-TAU: Specialist treatment as usual
TAU: Treatment as usual
TFP: Transference-focused psychotherapy
TiC-P: Trimbos and Institute for Medical Technology Assessment Questionnaire for Costs Associated with Psychiatric Illness
